# The impact of serum-free culture on HEK293 cells: From the establishment of suspension and adherent serum-free adaptation cultures to the investigation of growth and metabolic profiles

**DOI:** 10.3389/fbioe.2022.964397

**Published:** 2022-09-06

**Authors:** Mi Jang, Ellen Sofie Pete, Per Bruheim

**Affiliations:** Department of Biotechnology and Food Science, Norwegian University of Science and Technology, Trondheim, Norway

**Keywords:** HEK293, intracellular metabolite profiling, serum-free adaptation, central carbon metabolism, growth performance

## Abstract

Serum-free cultures are preferred for application in clinical cell therapy and facilitate the purification processes of bioproducts, such as vaccines and recombinant proteins. It can replace traditional cell culture - eliminating potential issues posed by animal-derived serum supplementation, such as lot to lot variation and risks of pathogen infection from the host animal. However, adapting cells to serum-free conditions can be challenging and time-consuming, and is cell line and medium dependent. In addition, the knowledge of the impact of serum-free culture on cellular metabolism is limited. Herein, we successfully established serum-free suspension and adherent cultures through two adaptation procedures for HEK293 cells in serum-free Freestyle 293 medium. Furthermore, growth kinetics and intracellular metabolic profiles related to central carbon metabolism were investigated. The entire adaptation procedure took 1 month, and high cell viability (>90%) was maintained throughout. The serum-free adherent culture showed the best growth performance, measured as the highest cell density and growth rate. The largest differences in metabolic profiles were observed between culture modes (adherent vs. suspension), followed by culture medium condition (control growth medium vs. serum-free medium). Metabolic differences related to the adaptation procedures were only seen in suspension cultures. Interestingly, the intracellular itaconate concentration was significantly higher in suspension cells compared to adherent cells. Furthermore, when the cells back-adapted from serum-free to serum-supplemented control medium, their metabolic profiles were immediately reversed, highlighting the effect of extracellular components on metabolic phenotype. This study provides strategies for efficient serum-free cultivation and deeper insights into the cellular responses related to growth and metabolism responses to diverse culture conditions.

## Introduction

Animal-derived serum is routinely used as a medium supplement for *in vitro* cell and tissue cultivation. The most common type is fetal bovine serum (FBS), which contains essential components*,* such as growth factors, attachment factors, vitamins, transport proteins, hormones, and trace elements that support the attachment, proliferation, and maintenance of a wide range of cells types *in vitro* (Brunner et al., 2010; van der Valk et al., 2018; Hesler et al., 2019). However, the use of FBS can be problematic in a scientific setting, as it has undefined composition, lot-to-lot variation, and the potential to cause unintended interactions with other substances. In addition, there is a risk of contamination with bovine viruses or *mycoplasma* that could affect the results and reproducibility *in vitro* cell culture system (Groothuis et al., 2015; Baker, 2016; van der Valk et al., 2018). Therefore, the production of vaccines and therapeutic proteins in the biopharmaceutical industry and biotechnology products for clinical purposes should be prepared in serum-free culture systems (Beltran Paschoal et al., 2014; Miki and Takagi, 2015).

Serum-free, chemically defined culture systems have the potential to improve the reproducibility in cell-based systems and can be formulated to reflect *in situ* human microenvironment more accurately (van der Valk et al., 2018). Notably, an increasing interest in primary cell culture is driving the shift to serum-free media culture (Depalma, 2016). An additional advantage of serum-free medium is that it facilitates a transition from adherent to suspension growth, which is crucial for scalability such as the bioreactor cultivation (Shridhar et al., 2017).

Unlike serum supplementation culture, which could be universally utilized for the cultivation of various cell types, serum-free culture is highly dependent on cell types and medium selection. Direct change to serum-free medium can lead to reduced cell viability (Rashid and Coombs, 2019) and proliferation due to the lack of many growth factors (Brunner et al., 2010). Therefore, going from serum-containing to serum-free cultures requires adaptation steps that progressively reduce serum concentration in the medium (Doyle and Griffiths, 1998; Biaggio et al., 2015). A key strategy for achieving successful serum-free culture is that the procedure provides sufficient time to adapt to the new medium conditions and those changes are gradually introduced (Beltran Paschoal et al., 2014). Nevertheless, the transition to serum-free culture can be challenging, labor-intensive, and time-consuming (Brunner et al., 2010; Caron et al., 2018). Most studies in this field have focused on the screening of diverse commercial serum-free media for the establishment of suspension culture and compared cell growth and production efficiency in CHO (Rodrigues et al., 2012, 2013; Jukić et al., 2016), and recently in Vero (Rourou et al., 2019), and MDCK cells (Wang et al., 2021). However, there are still no robust adaptation protocols available for the establishment of serum-free adherent cultures and the consequent transition to suspension culture mode.

The human cell line HEK293 cells have been widely utilized over the last decades, not only for the basic research such as protein interaction and signal transduction studies, but also for the production of recombinant proteins and viral vectors in biopharmaceutical research due to the high transfectivity, rapid growth, and human-like post-translation attributes (Petiot et al., 2015; Tripathi and Shrivastava, 2019; Tan et al., 2021). The HEK293 cell line is a naturally adherent type and is still typically cultured in serum-supplemented media. Its ability to grow in suspension culture in different commercial media has been reported. (Beltran Paschoal et al., 2014; Lomba et al., 2021). While HEK293 metabolism related to the vaccine or therapeutic protein production was extensively explored (Petiot et al., 2015), the information regarding the potential metabolic effects of serum-free culture and suspension growth is scarce. However, alterations in expression levels of genes involved in cholesterol biosynthesis have been reported for suspension derivatives of HEK293 (Malm et al., 2020), and Chinese hamster ovary (CHO) cells cultured in serum-free media displayed altered gene expression levels related to nucleotide synthesis and lipid metabolism (Shridhar et al., 2017). Furthermore, a recent study highlighted the effects of serum-free culture on immunostimulatory capacities, which correlated with enhanced catabolism of amino acids and glucose metabolism in dendritic cells (Calmeiro et al., 2021), suggesting that different medium compositions such as serum-free media influence cellular metabolome, with the potential of affecting their phenotypes and functional capacities.

Metabolomics is a superior technique for providing a comprehensive profile of metabolites and is widely acknowledged as the field of omics closest to the phenotype (Guijas et al., 2018). Previously, our group has reported changes in the central carbon metabolism - particularly the alteration of non-essential amino acids and pyruvate metabolism—in the C2C12 mouse muscle cell line when cultured in serum-free media, based on targeted metabolomics by mass spectrometry (Jang et al., 2022).

**FIGURE 1:**
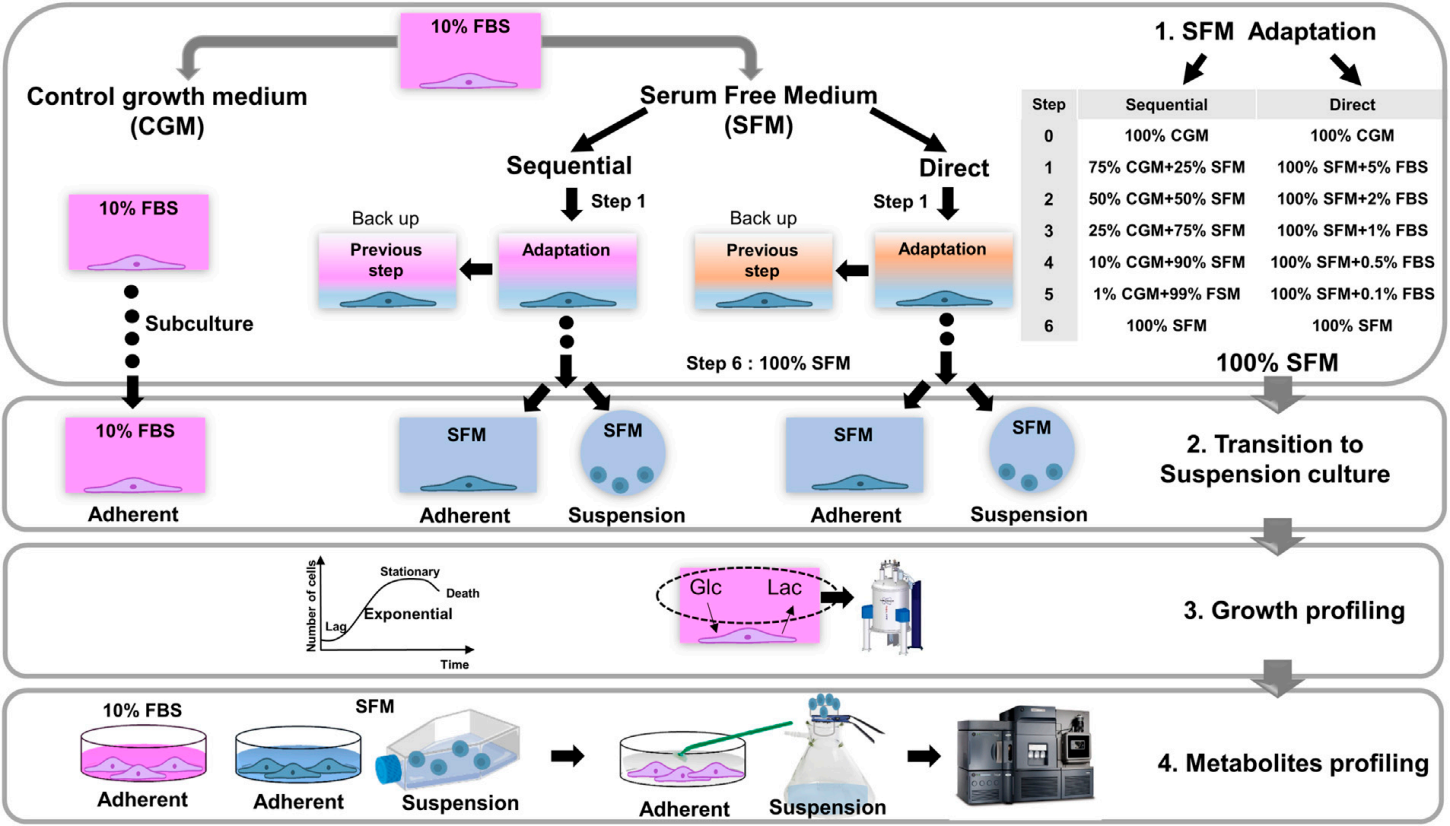
A Schematic workflow of the current study. 1) Strategies for the adaptation procedures of serum-free cultures of HEK293 cells by sequential and direct adaptations in Freestyle293 medium. The portion of serum is gradually reduced according to each step, described in Supplementary Table S1. Until reaching the 100% serum-free medium, cells were grown in adherent culture mode. During the proceeding of adaptation, backup cell samples were prepared in parallel under the same conditions as in previous step. 2) Establishment of final serum-free cultures for both adherent and suspension modes. The transition of adherent to suspension culture was performed at this stage. In total, 5 different culture systems were established (CGM; control growth medium culture*,* SFM Seq Ad; Serum-free medium sequential adapted adherent culture, SFM Dir Ad: Serum-free medium direct adapted adherent culture, SFM Seq Sus; Serum-free medium sequential adapted suspension culture, SFM Dir Sus; Serum-free medium direct adapted suspension culture), 3) After fully adapted serum-free culture, the growth of HEK293 cells were monitored in 5 different culture systems, and extracellular glucose and lactate in the medium were measured by NMR experiments. 4) Investigation of intracellular metabolic profiling. HEK293 cells under the exponential growth phase were harvested for the metabolic profiling analysis from 5 different culture systems. Adherent and suspension cells were extracted and targeted metabolomics based on mass spectrometry was applied to quantify the concentration of intracellular metabolites.

In this study, we described the strategies to establish successful serum-free adherent and suspension cultures. Adherent HEK293 cells were adapted to grow in a serum-free medium (Freestyle 293 expansion medium) following two different procedures and established serum-free suspension cultures from these cells. We further investigated the impact of adaptation procedures, serum-free medium, and culture modes on cell growth and intracellular metabolite profiles related to central carbon metabolism (Figure 1). We believe that our established serum-free adaptation culture methodology can be applied to a variety of other cell types with clinical and biopharmaceutical applications.

## Materials and methods

### Cell cultivation and maintenance in control growth medium

HEK293 cells were purchased from the American-type culture collection (CRL-1573, ATCC, United States). Cells were maintained in tissue culture flask T75 (Surface-treated, VWR, United States) in a control growth medium (CGM) consisting of Minimum Essential Medium Eagle (MEM, M0325, Merck, Germany) supplemented with 10% Fetal Bovine Serum (FBS) and 1% penicillin-streptomycin (15140122, Thermo Fisher Scientific, United States) and incubated at 37°C in a humidified atmosphere with 5% CO_2_ (HERAcell 150i, Thermo Fisher, United States). Cells are subcultured when they reached 70–80% of confluence under the exponential phase. Briefly, cells were enzymatically dissociated by incubating Accutase (A1110501, Thermo Fisher Scientific, United States) at room temperature (RT) for 5–8 min, then inactivated Accutase by diluting only with MEM medium (1:3, v/v) without FBS. After centrifugation (900 rpm, 3 min, RT), the cell pellet was resuspended in desired medium and seeded in new flasks. Importantly, subculturing of CGM was performed in parallel to maintain the same passage number.

### Adaptation of adherent HEK293 cells to serum-free medium

The cells were adapted to Freestyle 293 Expression medium (12338018, Thermo Fisher Scientific, United States) according to the two protocols detailed in Supplementary Table S1. After three initial passages in CGM from HEK293 stock, adaptation for serum-free culture was started from the 4th passage. Cells were subcultured as described in the section above with medium composition corresponding to the first step of the respective adaptation procedure. Progressive subcultures were performed when culture confluence reached 70%, and in parallel with the CGM control. Importantly, the backup cell samples from the previous adaptation steps were prepared in parallel until sufficient viability in the new condition was ensured. The viability of less than 80% would have warranted a regression to the previous adaptation steps until the viability improved. From step 6, HEK cells were seeded at 3 × 10^5^ cells/ml of density in the T75 flask (Treated for Increased cell attachment, VWR, United States) for adherent culture. To establish the fully adapted serum-free culture, cells should be subcultured at least three to five times in a 100% of Freestyle 293 expression medium.

### Switching from adherent to suspension culture in serum-free medium

The transition to suspension culture was performed from adherent cell cultures at step 6 of adaptation. For the maintenance of suspension culture, the Freestyle 293 Expression medium was additionally supplemented with 0.2% of Anti-clumping agent (0010057AE, Thermo Fisher Scientific, United States) and 1% of Pluronic F68 anti-clumping solution (24040032, Thermo Fisher Scientific, United States). Cells were seeded at 1.5 × 10^5^ cells/ml in the tissue flask T75 or T25 (non-treated, VWR, United States) and incubated on the orbital shaker (80 rpm) at 37°C in a humidified atmosphere with 7% CO_2_. The cell pellet after discarding the supernatant following the centrifugation (900 rpm, 3 min, RT) was incubated with Accutase for 1 min to prevent clumping, before seeding in a new flask during the subculture process.

### Preparation of stock cells from serum-free culture

Cell stocks were prepared from the serum-free cultures in a mixture of 47.5% of cryopreservation serum-free medium (C6295, Merck, Germany), 47.5% conditioned serum-free medium, and 5% DMSO, and stored in liquid nitrogen.

### Cell morphology monitoring and cell counting

Cell morphology was observed with a bright-field phase-contrast microscope (Eclipse T_S_2, Nikon, Japan), and all images were captured with NIS-Element F 4.51 software. The cell number as well as their average volume and size were determined by a Moxi^Z^ automated cell counter equipped with type s cassettes (Orflo Technologies, United States).

### Measurement of the cell viability by the trypan blue exclusion

The viability was determined by trypan blue (A13262, ThermoFisher, United States) excursion method. Briefly, cells were incubated with 0.4% trypan blue (1:1, v/v) for 1 min and placed in the hemocytometer for the counting of viable and non-viable cells. As the dye cannot cross intact cell membranes, only dead cells can be stained with blue color. Final viability has calculated the percentage of the number of viable cells in total cells based on the previous publication (Strober, 2015).

### Monitoring cell growth after serum-free culture adaptation

Cell growth was monitored after three times subcultures in 100% of the serum-free medium to ensure that cells were fully adapted to serum-free medium and exhibited stable growth. Suspension and adherent cells were seeded in the T25 flask at 1.5 × 10^5^ and 3 × 10^5^ cells/ml, respectively. Viable cell concentrations were determined from an individual flask per condition every day for 1 week in batch cultivation. The doubling times (DT) were determined according to the following exponential growth equation regression: DT = T ln2/ln (Xe/Xb); where T is the incubation time, Xb is the cell number at the beginning of the incubation time and Xe is the cell number at the end of the incubation time (Roth, 2006; Torres-Guzmán et al., 2017). Specific growth rates µ (h^−1^) were calculated based on the following equation: µ (h^−1^) = (lnXn-lnXn-1)/tn-tn-1, where X represents the viable cell density per ml, t represents the time point of the exponential growth phase in hours (Rourou et al., 2019).

### Preparation of adherent and suspension cells extracts

Adherent cells were seeded in 90 mm culture plates (6.6 ml medium, 3 × 10^5^ cells/ml of the density) and 6 wells plates (1.325 ml medium, 3 × 10^5^ cells/ml) for the metabolic sampling and the measurement of cell density and the average of cell volume, respectively. Suspension cells were seeded in the T25 flask (6 ml medium, 1.5 × 10^5^ cells/ml). The cells were harvested 48 h after seeding when cultures were in the exponential growth phase. Sampling was performed as described in a previous publication from our group, with slight modification (Røst et al., 2020). During the sampling of the adherent cells, the culture plate was placed on ice. After discarding the cell medium, cells were quickly washed with 10 ml of cold 0.9% NaCl solution followed by 10 ml of cold MQ-H_2_O. 6.5 ml of cold MQ:ACN (1:1, v/v) was added to the cell culture plate, then cells were detached mechanically by scrapping, and cell solution was transferred into the 50 ml centrifugation tube. This last step was repeated once more to ensure the collection of all cells grown on the plate. In total, 13 ml of cell solution was immediately placed in the liquid nitrogen for the quenching and stored at −80°C. In addition, cell density and average cell volumes were measured using a Moxi^z^ cell counter machine from the 6 wells plate for adherent cultures and directly from each flask for the suspension cultures. The filtration system (Røst et al., 2020) was applied to harvest the suspension cells. Briefly, 2.5 ml of cell suspension was added to a pre-wetted hydrophilic PVDF membrane filter (SVLP04700, Merck, Germany) and filtrated by applying a -200 mbar vacuum pressure. After washing with 5 ml of cold 0.9% of NaCl and MQ, the membrane filter with cells was transferred into a 50 ml centrifugation tube pre-filled with 13 ml of cold MQ:ACN (1:1, v/v) solution. To avoid clogging, two filters were collected per flask. The samples were placed in liquid nitrogen for quenching.

### Preparation of intracellular metabolites extracts

Intracellular metabolites were extracted from the quenched cell suspension through triple freeze-thawing cycles between liquid nitrogen and cold water (< 4°C). During this extraction procedure, the tubes were placed in the ice-cold water bath and vortexed between each cycle. After centrifugation (4°C, × 4500 g, 10 min) to remove cell debris, 12 ml of supernatant was collected and immediately quenched in liquid nitrogen. Following completed freeze-drying, lyophilized intracellular metabolites were reconstituted in 500 µL of cold MQ-H_2_O. After centrifugation (4°C, × 4500 g, 10 min), the supernatant was transferred to spin-filter with 3 kDa cutoff. After centrifugation (4°C, × 14,000 g, 20 min) for removal of compounds with molecular weights exceeding 3 kDa, the extracted solutions were stored at −80°C for MS-based metabolites analysis.

### Measurement of extracellular metabolites

The concentration of extracellular glucose and lactate were measured from the culture medium according to the previous publication from our group (Jang et al., 2022). Briefly, 2 ml of the medium was collected every day for 1 week from individual three T25 flasks prepared for monitoring of cell growth. After brief centrifugation (4°C, 1000 rpm, 3 min), 1.5 ml of the supernatant medium was transferred to a new tube and immediately quenched in liquid nitrogen and stored at −20°C. After freeze-drying, it was reconstituted in 600 µL of deuterium oxide (d_2_O) and measured by NMR (Bruker AscentTM 400 MHz) based on “N PROF_1H” method. 70 mM of creatine solution was used as a quantification reference.

### Targeted MS-based intracellular metabolite profiling

Intracellular metabolite profiling was performed in a targeted metabolomics approach, based on a triple quadrupole mass spectrometer (Xevo TQ-XS, Waters Corporation, United States). Phosphorylated metabolites related to pentose phosphate pathways (PPP), glycolysis, and other sugar phosphates and TCA intermediates were quantified based on capillary ion chromatography (CapIC, ThermoFisher, United States)—triple quadrupole mass spectrometer (MS/MS) according to the previous publication from our group (Stafsnes et al., 2018). For the analysis of intracellular lactate and pyruvate, cells and standards were derivatized with 1-Ethyl-3-(3ʹ-dimethylaminopropyl) carbodiimide (EDAC) and o-benzylhydroxyl amine (o-BHA), whereas amino acids analysis were performed with derivatization of phenyl isothiocyanate (PITC) according to a previous publication (Røst et al., 2020). UPLC (Accunity I-class UPLC, Waters Corporation, United States) coupled with TQ-XS was used for the quantification and further instrumental parameters were described in a previous publication (Røst et al., 2020). U^13^C(^15^N) labeled isotope chemicals were obtained from Cambridge isotope laboratories.

### Back adaptation into the serum-supplemented culture from serum-free culture

Adherent cells fully adapted to serum-free medium (passage 19) were back-adapted to grow in serum-supplemented medium (MEM+10% FBS). Cell extract samples were prepared for metabolic profiling as previously described. In total, three different cultures were prepared. 1) the serum-supplemented culture that had never been adapted to serum-free medium (CGM), 2) serum-free medium culture (SFM), and 3) serum-supplemented culture that had been back-adapted from the serum-free medium (from SFM to CGM). Cells were harvested after one subculture of back adaptation (early stage, passage 20) and after 5th subcultures (late stage, passage 24), in parallel with CGM and SFM (Figure 7A).

### Data processing

TopSpin v4.1.1 (Bruker, United States) was used to process all NMR spectra. After Fourier transformation, phase, and baseline correction of the NMR spectra, extracellular glucose and lactate concentration were quantified based on ERETIC2 function, with creatine as the external standard. All processing of MS-based data was performed in the TargetLynx application manager of Masslynx 4.1 (Waters Corporation, United States). All response factors are corrected by the corresponding U^13^C(^15^N)-isotopologue. Absolute intracellular metabolites concentrations were normalized based on dilution factors during sample preparation and to cell density and average cell volume.

### Statistics

All results are expressed as the average ±standard deviation (SD). To perform the multivariate statistical analysis in Metaboanalyst (Xia et al., 2009), all data were normalized with log transformation and auto-scaling. The student’s t-test was performed to compare the average of the two groups. One-way ANOVA followed by Tukey HSD was applied for the posthoc test using SPSS version 27 for the comparison of multiple groups.

## Results

### Morphology and cell viability during the adaptation procedures

**FIGURE 2:**
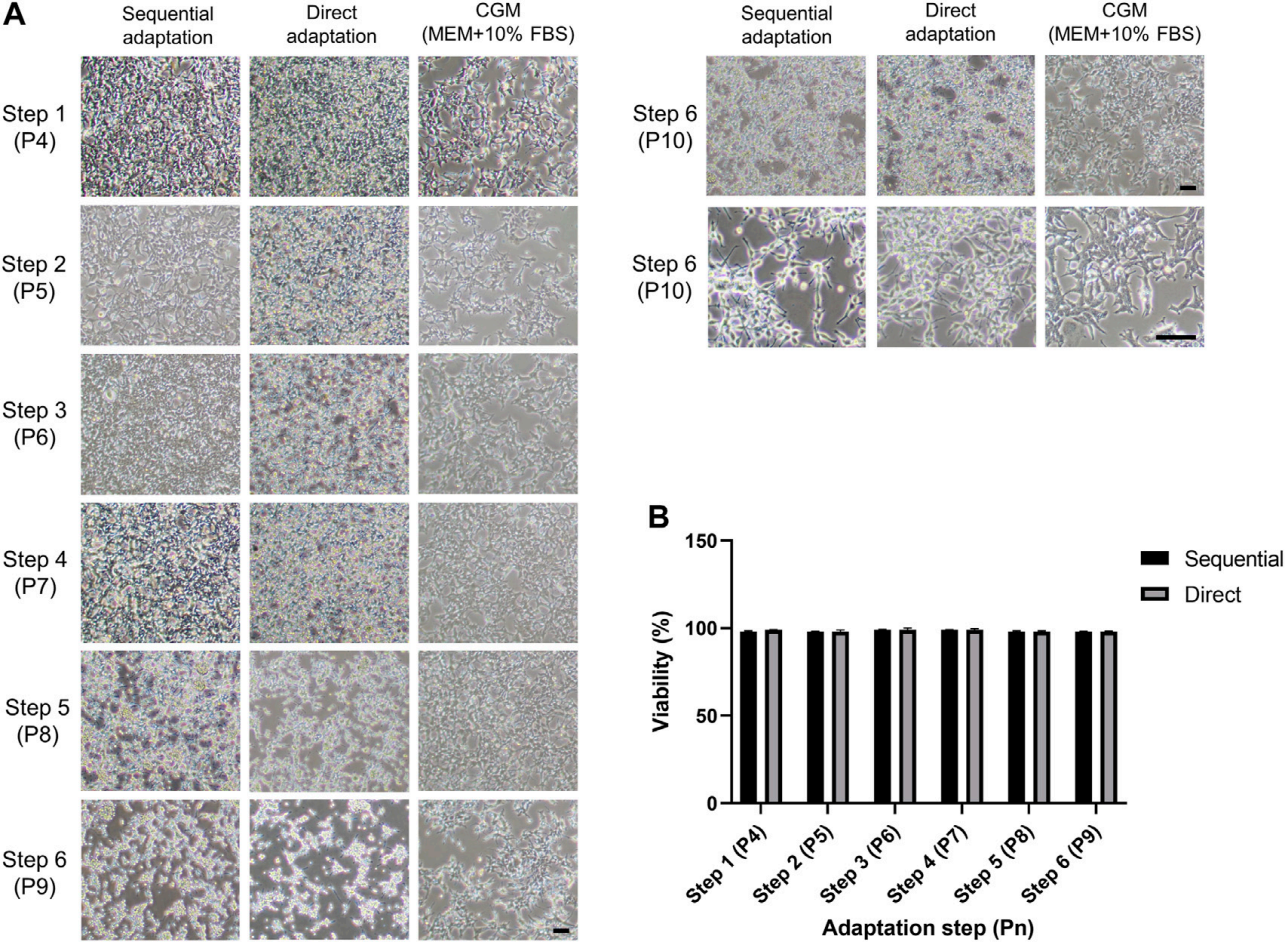
Morphology and cell viability of HEK293 cells during the adaptation procedures from step 1 to 6. **(A)** Phase-contrast images of each step in sequential and direct adaptations in SFM and in CGM. Left and right sides of the images showed the morphology of adherent cells cultivated in surface-treated and increased cell attachment flask, respectively. Scale bars indicate 100 µm. **(B)** Cell viability was measured during the entire adaptation steps. Pn indicates the passage number.

To efficiently establish serum-free culture with HEK293 cells, our strategy was to retain adherent culture with gradual reduction of the serum portion until reaching 100% serum-free medium. We applied two different stepwise procedures; sequential and direct (Supplementary Table S1). The cell viability should be maintained above 70–80% throughout the entire adaptation steps. In addition, cells can easily aggregate due to the decrease of serum portion, which is the biggest obstacle during the serum-free culture. Therefore, cell morphology and viability were determined at each step of the adaptation procedure. The phase-contrast images showed the morphology of HEK293 cells during sequential and direct adaptations (Figure 2A). The HEK 293 cells maintained their polygonal shape, characteristic of epithelial-like cells, for the first two steps of the adaptation procedures (Figure 2A, step 2). Interestingly, slight morphological changes indicative of a round shape were observed from step 3 in direct and step 4 in a sequential adaptation procedure (1% FBS). Over the course of the adaptation, the adherent cells became more rounder with slim membrane protrusions. At the last stage of adaptation, cells displayed reduced adhesion and detached easily (Figure 2A, step 6, P9). After changing from the surface-treated to the increased cell attachment flask for adherent culture, more stable adhesion was observed (Figure 2A, step 6 P9 to P10 Figure 2B). Viability below 70% would have warranted a reversion to the previous step of the procedure, but surprisingly, the cell viability was maintained above 90% throughout both adaptation procedures (Figure 2B) despite the morphological changes observed.

### Establishment of adherent and suspension serum-free cultures

**FIGURE 3:**
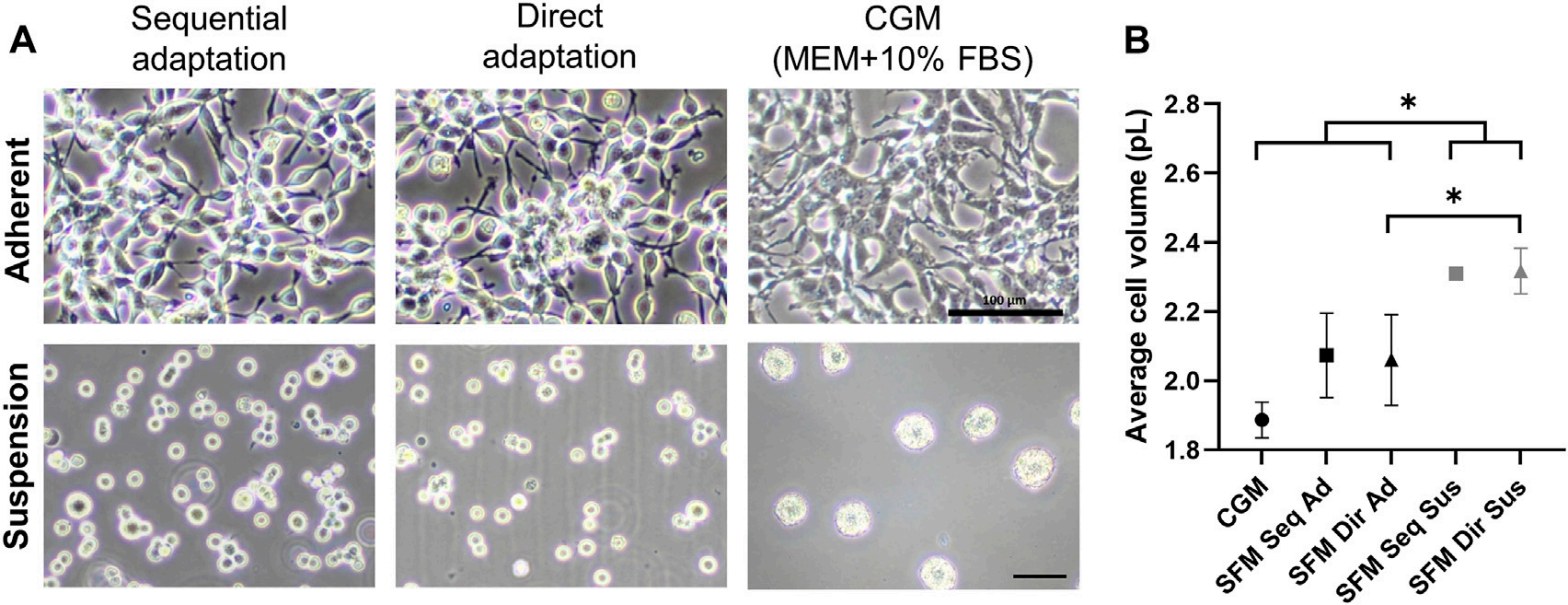
**(A)** Bright-field images of adherent and suspension HEK293 cells after completing serum-free medium cultures. Scale bars indicate 100um. **(B)** Cell volumes were measured on the second day in 5 different cultures. * indicates a significant difference between the groups (*p* < 0.05) after Tukey multiple comparisons test. (CGM; control growth medium culture*,* SFM Seq Ad; Serum-free medium sequential adapted adherent culture, SFM Dir Ad: Serum-free medium direct adapted adherent culture, SFM Seq Sus; Serum-free medium sequential adapted suspension culture, SFM Dir Sus; Serum-free medium direct adapted suspension culture).

After completing the entire adaptation procedure, the transition of culture mode from adherent to suspension was performed to establish serum-free suspension cultures. Adherent cells cultivated in the surface-treated flasks were transferred to the non-treated culture flasks and incubated on the orbital shaker (80 rpm) to initiate suspension culture. Anti-clumping solution was additionally supplemented to protect cell aggregates formation. After three more subcultures in serum-free medium supplemented with anti-clumping solutions, the culture consisted predominantly of single cells without clumping, indicating the successful achievement of suspension serum-free culture (Figure 3A). For serum-free adherent culture, an increased cell attachment flask should be used instead of the surface-treated flask, resulting in stable adhesion to the surface. Based on cell volume measurement, the suspension-adapted cells (2.31 ± 0.01 pL in SFM Seq Sus, 2.32 ± 0.07 pL in SFM Dir Sus) were significantly larger than the adherent ones (1.89 ± 0.05 pL in CGM, 2.07 ± 1.2 pL in SFM Seq Ad, 2.06 ± 1.3 pL in SFM Dir Ad) (Figure 3B). However, the establishment of suspension culture in the CGM (serum-supplemented medium) was impossible due to the formation of cell aggregates (Figure 3A). Therefore, a total of 5 different culture systems were compared for further study.

### Comparison of growth performance and extracellular glucose and lactate concentrations in 5 different culture systems

**FIGURE 4:**
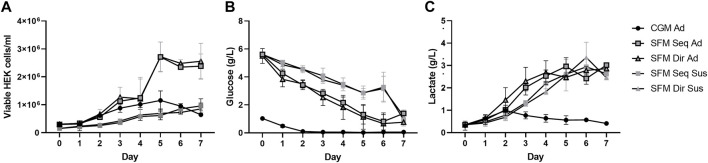
Cell growth was profiled for 7 days of batch cultivation in 5 different culture systems. **(A)** Cell growth kinetics, **(B)** Extracellular glucose, and **(C)** Lactate levels were measured every day of cultivation. All data represent average ±standard deviation. (*n* = 3).

The 5 different cultures were maintained in batch culture for 7 days, with daily monitoring of the number of viable cells, extracellular glucose, and lactate concentration. (Figure 4 and Table 1). SFM-adapted adherent HEK293 cells showed the highest viable cell density (3 × 10^6^ cells/ml), the shortest doubling time (32 h), and the highest growth rate (0.02 h^−1^) and remained the stationary phase without entering the death phase until 7 days of cultivation. In contrast, when CGM adherent HEK293 cells reached the maximum cell density (1.2 × 10^6^ cells/ml), it immediately entered the death phase on the same day without maintaining a stationary phase. No death phase was observed in suspension cultures, which reached a similar viable cell density (1 × 10^6^ cells/ml) as the CGM adherent culture. In addition, similar doubling time (43–48 h) and specific growth rates (0.014–0.016 h^−1^) were also observed between CGM and SFM suspension cultures. No significant differences in growth profiles were observed between different adaptation procedures.

**TABLE 1:** Growth profiles and Biochemical analysis of the 5 different culture systems. Data not sharing a common superscript alphabetic letter indicate significant difference (*p* < 0.05) after Tukey posthoc test for multiple comparisons.

	CGM	SFM Seq Ad	SFM Dir Ad	SFM Seq Sus	SFM Dir Sus
Maximum cell density (cells/ml)	1.1–1.4 × 10^6^	2.3 × 10^6^–3.2 × 10^6^	2.6 × 10^5^–3.3 × 10^6^	7 × 10^5^–1.1 × 10^6^	7 × 10^5^–1.0 × 10^6^
Doubling time, (h)	43 ± 4^a^	32 ± 3^b^	34 ± 5^b^	48 ± 2^a^	44 ± 5^a^
Specific growth rate (µ) (h^−1^)	0.016 ± 0.001^a^	0.020 ± 0.003^b^	0.021 ± 0.002^b^	0.014 ± 0.0006^a^	0.016 ± 0.001^a^
qGlc (pmol/cell/day)	3.19 ± 0.25	2.96 ± 0.33	3.31 ± 0.73	5.00 ± 0.64	4.81 ± 0.89
qLac (pmol/cell/day)	5.24 ± 1.06	4.34 ± 0.46	4.68 ± 0.94	9.4 ± 1.01	9.02 ± 2.47
YLac/Glc (qLac/qGlc)	1.62 ± 0.20	1.47 ± 0.01	1.42 ± 0.03	1.95 ± 0.50	2.07 ± 1.03

A higher initial glucose concentration was measured in the SFM (5.5 g/L) than in the CGM (1.0 g/L) (Figure 4B). The glucose concentration in all culture systems decreased over time. However, higher consumption of glucose (at day 1) was seen in direct than in sequential adapted cells. In the CGM culture, the glucose was depleted on day 2, whereas the SFM cultures never reached complete depletion. Similar maximum levels of lactate (3 g/L) were observed on the 5th day in adherent and 6th days in suspension culture of SFM, respectively, before the accumulation stagnated (Figure 4C). Then, no more accumulation was observed. Interestingly, the lactate concentration increased until day 2 before it started to decrease, suggesting that HEK293 consumed the lactate after glucose depletion. The specific rates for the consumption of glucose, production of lactate, and yield of lactate production from glucose consumption are summarized in Table 1. In batch culture, the specific glucose consumption rate (qGlc) and lactate production rate (qLac) in suspension cultures are higher compared to that in adherent culture. However, the ratio of glucose and lactate (*Y*Lac/Glc) does not show any difference between all culture systems (*p* = 0.703), indicating that 5 different culture systems stably maintained central carbon metabolism regulated by glucose and lactate.

### Comprehensive analysis of intracellular metabolic profiles in 5 different culture systems

**FIGURE 5:**
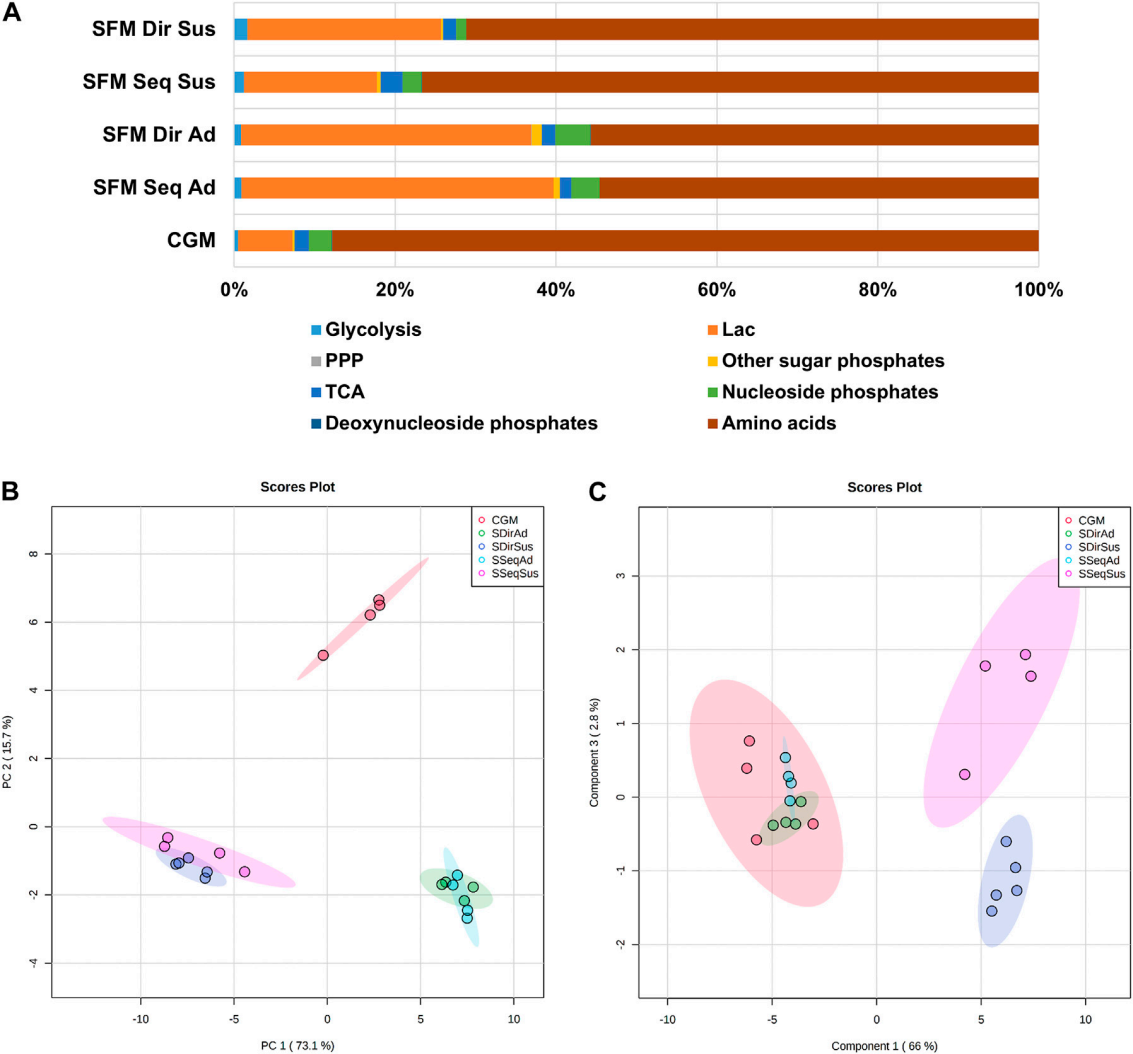
The overview of intracellular metabolite profiles in 5 different culture systems. **(A)** The contribution (%) of metabolic classes to the total measured intracellular metabolites. **(B)** 2D score plot of Principal Component Analysis (PCA) and **(C)** Partial Least Squares-Discriminant Analysis (PLS-DA) with total 61 metabolites in 5 different culture systems. The colored ellipses represent the 95% confidence interval of the sample grouping. After log transformation and autoscale normalization, multivariate statistical analysis was performed in MetaboAnalyst. (CGM; control growth medium culture; red-circles*,* SSeqAd; Serum-free medium sequential adapted adherent culture; light-blue circles, SDirAd: Serum-free medium direct adapted adherent culture; green-circles, SSeqSus; Serum-free medium sequential adapted suspension culture; pink-circles, SDirSus; Serum-free medium direct adapted suspension culture; blue-circles).

After characterization of cell growth in all culture systems, cells were collected during the exponential growth phase for intracellular metabolite profiling. In total, 61 metabolites involved in the central carbon metabolism were quantified (Supplementary Table S2). The absolute concentrations of most metabolites were lower in suspension cells compared to adherent cells, except itaconate (IA) and succinate. (Supplementary Figure S1 and Supplementary Table S3). The contribution of different metabolic pathways to the total metabolite pools was calculated for each culture, revealing groupwise differences (Figure 5A). SFM cultures showed an increased contribution of lactate (31–38% in adherent and 16–24% in suspension) compared to CGM (7%), while the contribution of amino acids was decreased (55% in SFM adherent 70–77% in SFM suspension versus 88% in CGM). Nucleoside phosphates contributed more to the total metabolite pools in the adherent cultures (2.8% in CGM, 3.5–4.3% in SFM adherent) than in the suspension cultures (1.2–2.4%).

We subsequently performed multivariate statistical analysis to explore the similarities or differences in the entire set of metabolites between the 5 different cultures. First, principal component analysis (PCA) (Worley and Powers, 2013), which is an unsupervised algorithm providing unbiased dimensionality reduction, was performed to obtain general information about the datasets, relationships between the different groups, and detection of outliers (Kumar et al., 2020) (Figure 5B). The score plot of two principal components (PC1; first principal component and PC2; second principal component) accounts for 88% of the total variance from PCA of 61 metabolites datasets (Figure 5B). Culture modes between adherent and suspension showed more distinctive metabolic differences than the medium type, since PC1 (describing 73.1% of total variance) covered more variance than PC2 (describing 15.7% of total variance). Interestingly, there is a slight separation between sequential and direct adaptation only in suspension culture, but not in the SFM adherent cultures. We further subsequently performed supervised PLS-DA (partial least squares discriminant analysis) with Y-variables (5 different culture groups), confirming the observations from the PCA score plot, and clearly demonstrating a separation between suspension cultures derived from the two different adaptation procedures (Figure 5C). The cross-validation scores for the model were R2 = 0.96 and Q2 = 0.65, which is an acceptable value of ≥0.4 in the case of the biological models (Worley and Powers, 2013), indicating that this PLS-DA model is valid. Moreover, there was both a medium-dependent and culture mode-related difference in the amino acid pool, but the nucleotide pool was mostly affected by culture modes (Supplementary Figure S2). Taken together, the most distinct differences in intracellular metabolites related to central carbon metabolism were observed between culture modes (Adherent vs. Suspension), followed by media nutritional effects (SFM vs. CGM) and adaptation procedures (sequential vs. direct).

### Comparison of intracellular metabolic profiles between culture modes, media types, and adaptation modes

**FIGURE 6:**
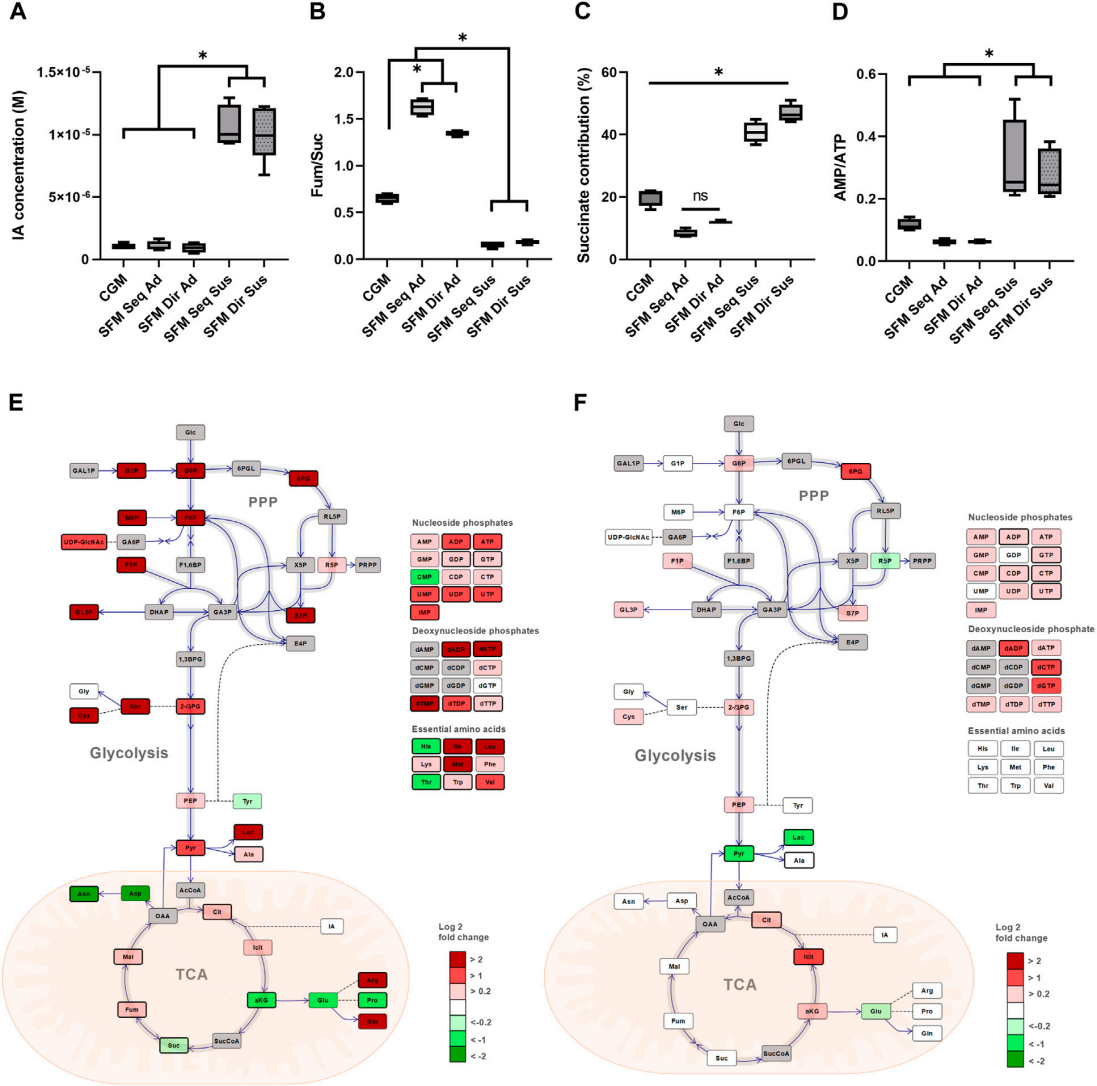
Comparison of alteration of intracellular central carbon metabolism between different sub-groups. Comparison between Adherent and Suspension culture. **(A)** Absolute intracellular concentration (M) of IA. **(B)** The ratio of fumarate/succinate. **(C)** The contribution of succinate in total TCA cycle intermediates pool. **(D)** The ratio of AMP/ATP in 5 different culture systems. Simplified schematic alteration of central carbon metabolism in HEK293 cells with a heatmap of the log2 fold change of average metabolites levels. Dark gray color metabolites were not determined. Direct and indirect reactions are shown by continuous lines and dashed lines, respectively. TCA; tricarboxylic acid, PPP; pentose phosphate pathway, **(E)** Comparison between SFM and CGM adherent culture. The log2 fold change of average of SFM/CGM was shown. SFM sequential and direct-adapted adherent cultures were merged. **(F)** Comparison between sequential and direct adaptation in SFM suspension culture. The log2 fold change of average of SFM Seq Sus/SFM Dir Sus was shown. Statistically significant different metabolites (*p* < 0.05) were illustrated with a bold border and the graphs were illustrated using OMIX software^55^. Data presented as average ±standard deviation.

As the multivariate analysis revealed the subgroup-dependent differences, we further investigated which intracellular metabolites contributed to the distinction. First, the endometabolome set of adherent and suspension cells was compared. As previously mentioned, most metabolites were significantly lower in suspension than in adherent culture. The exception was itaconate (IA), which was present at significantly higher levels in suspension than in adherent cells (*p* < 0.001) (Figure 6A). The previous publication has reported that IA inhibits SDH (Succinate dehydrogenase) in mitochondria, resulting in the accumulation of succinate (Lin et al., 2021). Therefore, we further calculated the ratio of fumarate to succinate (Fum/Suc) to determine whether IA could inhibit SDH. Surprisingly, the ratio of fumarate to succinate was significantly lower in suspension cultures (0.16–0.18 in suspension, 0.6–1.6 in adherent), indicating less conversion of succinate to fumarate (Figure 6B). Next, the succinate’s contribution to the TCA cycle-related metabolite pool was calculated, demonstrating a suspension culture-specific accumulation of the metabolite (fold change 2-4, compared to the adherent culture) (Figure 6C). While there were less of all nucleoside phosphates in suspension cultures, AMP to ATP ratio (AMP/ATP) was significantly higher (fold change 3–5) in these cultures than in the adherent ones (Figure 6D).

Secondly, the effects of culture media were investigated by comparing the central carbon metabolism of the adherent cultures in CGM (MEM +10% FBS) and SFM (Freestyle293 media). For this purpose, SFM sequential and direct adaptation adherent cultures were merged, as our previous results indicated that they were metabolically similar. The simplified central carbon metabolism of the SFM relative to CGM in adherent culture was visualized (Figure 6E), revealing higher concentrations of glycolytic-related metabolites, PPP intermediates, and lactate in SFM than in CGM adherent culture.

Lastly, the effects of different adaptation procedures on suspension cells were compared with regard to the central carbon metabolism (Figure 6F). Significant changes in the amino acid pools were not observed by two different adaptation procedures in suspension culture. However, the level of pyruvate and lactate were significantly lower, while the level of citrate and isocitrate in the TCA cycle were significantly higher in sequential versus direct adapted suspension culture.

### Back adaptation from the serum-free to control growth medium supplemented with serum culture

**FIGURE 7:**
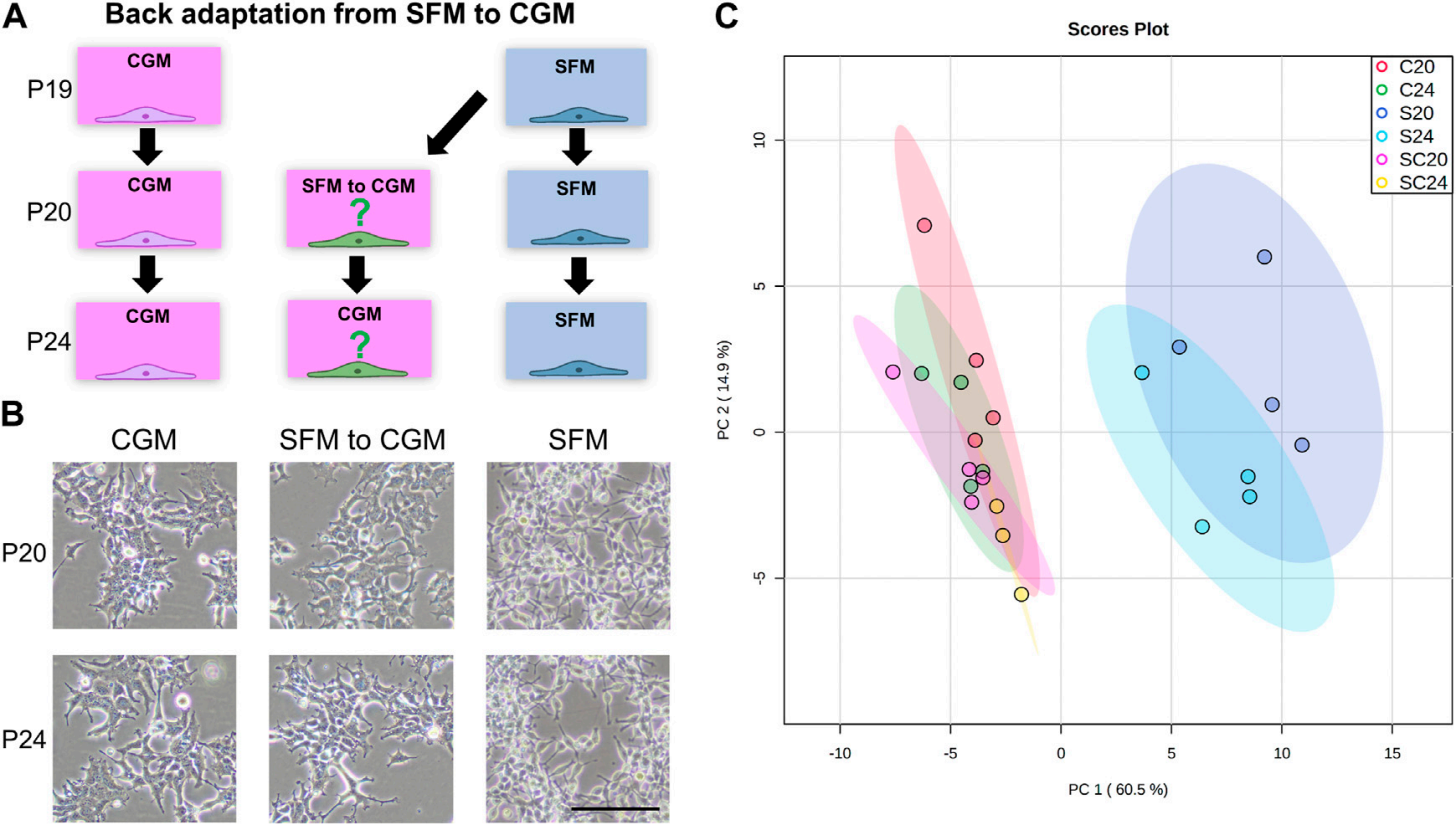
**(A)** Back adaptation procedure from SFM (serum-free medium, Freestyle293) to CGM (control growth medium, MEM+10% FBS). Passage 19 of HEK293 cells (Fully SFM-adapted adherent cells) was initiated for the back adaptation. Early (after one subculture, passage number 20, P20) and late-stage (after 5 times subculture in CGM, passage number 24, P24) of back adaptation were monitored, and the same passage number of HEK293 cells cultured in CGM and SFM were also prepared in parallel. **(B)** Representative bright-field images were shown to observe cell morphology after 48 h of cultivation. The scale bar indicates 100 µm. **(C)** 2D score plot of PCA analysis of whole metabolome sets of 6 different groups was shown. C, S, and SC indicate CGM, SFM, and SFM to CGM culture, respectively. The color ellipse in the score plot illustrates the 95% confidence regions.

To examine whether the metabolic phenotypes of the serum-free cultures were permanently altered, we adapted the adherent SFM cultures back to CGM (Figure 7A). Representative morphological images in the early (P20) and late stage (P24) of back adaptation are shown in Figure 7B. After just one passage of back adaptation in CGM, the cells displayed morphologies similar to that already seen in CGM. We further profiled intracellular metabolites and the final PCA score plot was shown in Figure 7C. Back-adapted cultures from SFM to CGM clustered together with CGM culture, only SFM was classified separately. Surprisingly, there is no separation between the early and late stage of back adaptation, indicating that metabolic phenotypes of the cultures were immediately affected by the medium composition.

## Discussion

In this study, we successfully established adherent and suspension cultures of HEK293 cells in Freesytle 293 serum-free medium, through two different adaptation approaches. Furthermore, we investigated the impact of serum-free culture on cell growth and central carbon metabolism profiles, providing new insights regarding the effects of culture media (CGM vs. SFM), culture modes (adherent vs. suspension), and adaptation approaches (sequential vs. direct). To the best of our knowledge, this is the first study to compare different biological aspects after achieving serum-free cultures from a conventional serum-supplemented culture in HEK293 cells.

During the adaptation, slight changes in morphology were first observed when the serum concentration is 1% (step 3 in direct adaptation, step 4 in sequential adaptation), suggesting that 1% of serum concentration is a critical step before reaching a 100% serum-free medium culture (Figure 2A). 2% of FBS could be proposed as a starting point for the adaptation procedure to save time. When in 100% serum-free medium, the adherent cells were easily detached, but changing from surface-treated to increased cell attachment flasks solved this problem (Figure 2A step 6 P9 to P10). Cell viability overall remained high (>90%) (Figure 2B), indicating that the slightly altered morphology was not a sign of cell distress. From an industrial perspective avoiding cell clustering in suspension cultures is critical, as the consequent oxygen limitation can drastically reduce viability and productivity (Petiot et al., 2015). However, the formation of cell aggregates was prevented by additional supplementation of anti-clumping solution as well as resuspension in Accutase at each subculture step, resulting in stable growth as a single-cell suspension (Figure 3). However, when this procedure was applied in CGM the cells still clustered, suggesting that serum-free culture is the best fit for cells grown in suspension. Our serum-free culture systems were established within 1 month, which is faster than the previous serum-free culture performed for 6 months (Rodrigues et al., 2013). Another study reported that 27 days are required for the establishment of rapid serum-free cultures of CHO cells (Wu et al., 2021), indicating that the timeframe of our adaptations is reasonable. Interestingly, the average volumes of our suspension cells were increased compared to adherent cells (Figure 3B). A similar phenomenon has been reported for MDBK cells after the transition to serum-free suspension culture. Limitation of growth space and contact inhibition might be affecting the size of the cells, whilst the removal of these restrictions results in larger cells in suspension (Seirin Lee, 2016; Wang et al., 2021). No differences related to cell morphology, viability, or size were observed between the two adaptation procedures, but the direct approach is slightly more time- and cost-efficient as it does not require the preparation of two types of media.

Taken together, not only adherent but also suspension culture in serum-free Freestyle293 medium were successfully established according to our strategies. Given that adherent cells have traditionally been widely used for the production of viruses and clinical research, as well as that suspension growth is the platform of choice for bioproduction of therapeutic proteins, the establishment of both culture modes in a serum-free medium could be useful for the wide range of application fields. Our adaptation protocols may serve as templates for others seeking to establish serum-free cultures.

Among our culture models, the SFM adherent culture showed the best growth performance (Figure 4A; Table 1). Previous studies with HEK293 have reported similar maximum cell densities in animal origin-free medium (up to 3 × 10^6^cells/ml and DT = 24 h) (Cervera et al., 2011) and in serum-free EX-CELL 293 medium (2 × 10^6^ cells/ml, DT = 33 h) (Sahni et al., 2012). Although SFM suspension cultures were seeded at half density of the adherent cultures, they reached the same density as the CGM culture (1 × 10^6^ cells/ml). Moreover, we have tested higher cell seeding density to increase the maximum cell density in suspension culture, however, it resulted in lower growth rates (data not shown).

Lactate is often described as a toxic metabolic waste product, and its accumulation in mammalian cell cultures can cause changes in pH and osmolality that in turn inhibit cell growth (Pereira et al., 2018). Higher extracellular levels of lactate were measured in the SFM culture than in the CGM culture, possibly due to the difference in initial glucose concentrations in the medium (5.5 g/L in SFM versus 1 g/L in CGM) (Figure 4B). The maximum lactate levels at the late stage of both the adherent and suspension cultures exceeded 20 mM (2 g/L), which in some cases inhibits cell proliferation (Shen et al., 2019). Nevertheless, the cell densities reached in our SFM cultures (1–3 × 10^6^ cells/ml) were well within the range of what is needed when HEK293 cells are used in the viral production (1–4 × 10^6^ cells/ml) (Petiot et al., 2015). Previous publications have also shown a similar specific consumption rate of glucose (3–4 pmol/cell/day) and production rate of lactate (3.5–4.5 pmol/cell/day) (Zhan et al., 2020), as well as the ratio of Lac/Glc (1–2.35) (Petiot et al., 2015) in other HEK293 culture systems (Table 1). Moreover, our results indicated the consumption of lactate by the cells. In fact, lactate could be consumed when glucose is depleted or when cells enter the stationary phase mostly performing oxidative phosphorylation (Leong et al., 2017; Ritacco et al., 2018). It might be explained that lactate consumption occurred due to glucose depletion and entering a stationary phase in the case of CGM (day 2) and SFM cultures (day 5 for adherent and day 6 for suspension), respectively (Figures 4B,C). Interestingly, there seems to be a positive link between the lactate consumption phenotype and capacity for protein production (Pereira et al., 2018). These features could potentially be explored to enhance protein production efficiency in fed-batch or perfusion culture systems.

Analysis of the intracellular metabolite profiling data identified culture mode (adherent vs. suspension) as the most prominent driver of group separation, followed by medium types (CGM vs. SFM). However, only suspension cultures showed different metabolic phenotypes between adaptation procedures (sequential vs. direct) (Figures 5B,C).

Regarding a comparison of culture modes, most intracellular metabolites were significantly lowered in suspension compared to adherent culture. A similar observation was recently reported that, compared to their adherent progenitors, suspension HEK293 cell lineages have downregulated gene expression related to oxidative phosphorylation, nucleotide, and amino acids metabolism except for cholesterol metabolism by genomic, transcriptomic, and metabolic gene analysis (Malm et al., 2020). Moreover, proteomics analysis showed down-regulated enzyme activity or decreased peptide intensity related to glycolysis, PPP, and partially TCA cycle pathway in the exponential growth phase of suspension compared to adherent culture in MDCK cells (Pech et al., 2021).

Unexpectedly, the concentration of itaconic acid (IA) was significantly higher in suspension than in adherent cultures (Figure 6A). IA is produced from cis-Aconitate by aconitate decarboxylase 1, encoded by the *Irg1* gene. As IA inhibits succinate dehydrogenase (SDH), the enzyme converting succinate to fumarate, high levels of IA can result in the accumulation of succinate (Lin et al., 2021). Our results also confirmed the significant accumulation of succinate in the total TCA cycle pool as well as the decrease in the ratio of fumarate/succinate only in suspension culture, potentially due to IA-induced SDH inhibition (Figures 6B,C). To the best of our knowledge, this is the first report to observe the accumulation of IA in suspension cells. Interestingly, few previous studies reported the link between Irg1 and EMT (Epithelial-Mesenchymal transition), involving loss of epithelial and acquisition of mesenchymal properties. Overexpression of *Irg1* showed upregulated gene expression related to EMT, resulting in increased growth and invasion in glioma cells (Pan et al., 2014). MicroRNA-378, for which Irg1 is a direct target, inhibits cell migration, invasion, and EMT in human glioma in human glioma (Shi et al., 2018). Furthermore, SDH knockdown enhanced proliferation and EMT in mouse ovarian cancer cells (Aspuria et al., 2014). Our results also demonstrated an increased ratio of AMP/ATP only in suspension culture, indicating potential involvement of AMPK activation (Figure 6D). Interestingly, a previous study reported that activated AMPK can promote EMT, while inactivation of AMPK induced re-attachment in multiple types of cancer cells (Saxena et al., 2018). In addition, a recent publication detected a 4-fold increase in the ratio of AMP/ATP in MDCK cells when transitioned from adherent to suspension culture, which is in accordance with our culture models. They further postulated that it could implicate the involvement of the AMPK pathway (Pech et al., 2021). Therefore, the link between EMT mechanisms mediated by IA, SDH, and AMPK pathways could be further investigated to understand the more detailed metabolism of a transition from adherent to suspension cells.

Interestingly, nucleotide pools were affected by the culture mode (adherent vs. suspension) rather than by different media (CGM vs. SFM), whereas AA pools could be influenced by both factors of culture modes and the different media (Supplementary Figure S2). An explanation might be that proliferating cells are highly dependent on endogenous production of nucleotides because their direct uptake from the extracellular space is negligible, while they often rewire the AA metabolism to actively acquire amino acids from the extracellular space to maintain biomass accumulation (Zhu and Thompson, 2019).

HEK293 cell metabolism is reportedly skewed towards aerobic glycolysis, and most of the pyruvate is not used for cellular energy generation through the TCA cycle but is converted to lactate (Petiot et al., 2015). Our results showed that SFM culture favored aerobic glycolytic energy metabolism rather than mitochondrial energy metabolism, based on the high levels of glycolytic intermediates and lactate (Figure 5A and Figure 6E). Among our cultures, proliferation was most prominent in the SFM adherent culture, likely due to high PPP pools and the subsequent increase in the production of nucleotide pools. Moreover, suspension culture showed different metabolic profiles between different adaptation approaches. Sequentially adapted cells seemed to rely more on mitochondrial metabolism than directly adapted cells (Figure 6F). This could mean that sequential adapted suspension cells still preserve the metabolic properties seen in CGM, such as a similar contribution of lactate, TCA cycles, and AA to the central carbon metabolism. Hence, proper suspension adaptation procedures could be chosen according to the intended use of the cells for industrial and research purposes.

Lastly, back-adaption of adherent cells from SFM to CGM immediately reverted their metabolic profiles to the original state, indicating that cellular metabolism was not altered permanently by serum-free cultures and could be immediately influenced by extracellular nutrient compositions (Figure 7). This suggests that the metabolic phenotype of HEK293 cells is tightly regulated by extracellular nutrients—a trait that could be of industrial value.

## Conclusion

We successfully established serum-free adherent and suspension cultures for HEK293 cells throughout two different adaptation procedures (Sequential and direct) in the serum-free medium (Freestyle 293 medium). The entire procedure took approximately 1 month. SFM adherent culture demonstrated improved growth performance than CGM culture, while SFM suspension cultures were comparable to CGM adherent culture. However, the rate of lactate production and glucose consumption was similar in all culture systems. Interestingly, culture mode (adherent vs. suspension) was a bigger driver of the central carbon metabolism alterations than the medium composition (SFM vs. CGM). Moreover, significant accumulation of IA and succinate, and an increased ratio of AMP/ATP were observed in suspension versus adherent cells. Our SFM-adapted adherent cells facilitate aerobic glycolytic energy metabolism for supporting cell growth. Interestingly, the metabolic profiles of the sequential- and direct-adapted suspension cultures were different, with the former being more dependent on mitochondrial metabolism than the latter. Finally, the metabolic phenotype of the adherent SFM culture was not maintained when reverted to CGM. We believe that our methodology for establishing serum-free adherent and suspension cultures, combined with new deeper insights into the cellular response to the various cultivation factors (different culture modes, media, and adaptations), could be useful for diverse industry and research applications.

## Data Availability

The original contributions presented in the study are included in the article/Supplementary Material, further inquiries can be directed to the corresponding author/s.
