# Supplementary material for: The impact of serum-free culture on HEK293 cells: From the establishment of suspension and adherent serum-free adaptation cultures to the investigation of growth and metabolic profiles
Source: Front Bioeng Biotechnol. 2022 Sep 6;10:964397. doi: 10.3389/fbioe.2022.964397 (PMC9485887; doi:10.3389/fbioe.2022.964397)
Supplement: Supplementary file 1 [file Table1.DOCX]

Supplementary Material

**Supplementary Table 1.**  Each step for two different adaptation procedures (Sequential and Direct adaptations) of HEK293 cells to adapt in serum-free medium (SFM).

| Steps | Sequential adaptation | Direct adaptation |
| --- | --- | --- |
| 0 | 100% CGM | 100% CGM |
| 1 | 75% CGM +25% SFM | 100% SFM + 5% FBS |
| 2 | 50% CGM + 50% SFM | 100% SFM + 2% FBS |
| 3 | 25% CGM + 75% SFM | 100% SFM + 1% FBS |
| 4 | 10% CGM + 90% SFM | 100% SFM + 0.5% FBS |
| 5 | 1% CGM + 99% SFM | 100% SFM + 0.1% FBS |
| 6 | 100% SFM | 100% SFM |

**Supplementary Table 2.** List of 61 metabolites abbreviations for the current study.

| **Class** | **Abbreviation** | **Full name** |
| --- | --- | --- |
| Glycolysis | G6P | Glucose-6-phosphate |
|  | F6P | Fructose-6-phosphate |
|  | 2/3-PG | 2-/3-Phopho-D.glycerate |
|  | PEP | Phosphoenolpyruvic acid |
|  | Pyr | Pyruvic acid |
| Lac | Lac | Lactate |
| PPP | 6PG | 6-Phospho-D-Gluconate |
|  | R5P | D-Ribose-5-phosphate |
|  | S7P | D-Sedoheptulose-7-phosphate |
| Other sugar phosphates | F1P | Fructose-1-phosphate |
|  | G1P | Glucose-1-phosphate |
|  | GL3P | Glycerol-3-phosphate |
|  | M6P | mannose-6-phosphate |
|  | UDP-GlcNAc | Uridine diphosphate *N*-acetylglucosamine |
| Nucleoside phosphates | AMP | Adenosine monophosphate |
|  | ADP | Adenosine diphosphate |
|  | ATP | Adenosine triphosphate |
|  | CMP | Cytidine monophosphate |
|  | CDP | Cytidine diphosphate |
|  | CTP | Cytidine triphosphate |
|  | GMP | Guanosine monophosphate |
|  | GDP | Guanosine diphosphate |
|  | GTP | Guanosine triphosphate |
|  | IMP | Inosine monophosphate |
|  | UMP | Uridine monophosphate |
|  | UDP | Uridine diphosphate |
|  | UTP | Uridine triphosphate |
| Deoxy nucleoside phosphates | dADP | Deoxyadenosine diphosphate |
|  | dATP | Deoxyadenosine triphosphate |
|  | dCTP | Deoxycytidine triphosphate |
|  | dGTP | Deoxyguanosine triphosphate |
|  | dTMP | Deoxythymidine monophosphate |
|  | dTDP | Deoxythymidine diphosphate |
|  | dTTP | Deoxythymidine triphosphate |
| TCA cycle | Cit | Citrate |
|  | Icit | Isocitrate |
|  | aKG | α-ketoglutarate |
|  | Suc | Succinate |
|  | Fum | Fumarate |
|  | IA | Itaconic acid |
|  | Mal | Malate |
| Amino acids | Ala | Alanine |
|  | Arg | Arginine |
|  | Asn | Asparagine |
|  | Asp | Aspartate |
|  | Cys | Cysteine |
|  | Gln | Glutamine |
|  | Glu | Glutamate |
|  | Gly | Glycine |
|  | His | Histidine |
|  | Ile | Isoleucine |
|  | Leu | Leucine |
|  | Lys | Lysine |
|  | Met | Methionine |
|  | Phe | Phenylalanine |
|  | Pro | Proline |
|  | Ser | Serine |
|  | Thr | Threonine |
|  | Trp | Tryptophan |
|  | Tyr | Tyrosine |
|  | Val | Valine |

**Supplementary Table 3.** The log 2 fold change of adherent/suspension cultures of absolute intracellular metabolites concentration. The three adherent (the CGM, SFM Seq Ad, and SFM Dir Ad) and two suspension cultures (SFM Seq Sus and SFM Dir Sus) were merged. (* P<0.05, **P<0.01, ***P<0.001)

| **Class** | **Abbreviation** | **Log2 Fold change**  **(Average of Adherent/Suspension)** |
| --- | --- | --- |
| Glycolysis | G6P | 4.448** |
|  | F6P | 4.102** |
|  | 2/3-PG | 5.562*** |
|  | PEP | 3.243*** |
|  | Pyr | 1.980** |
| Lac | Lac | 3.567** |
| PPP | 6PG | 2.375** |
|  | R5P | 3.418*** |
|  | S7P | 4.832** |
| Other sugar phosphates | F1P | 3.639* |
|  | G1P | 3.373** |
|  | GL3P | 5.031** |
|  | M6P | 5.421** |
|  | UDP-GlcNAc | 3.889*** |
| Nucleoside phosphates | AMP | 3.440*** |
|  | ADP | 3.244*** |
|  | ATP | 3.965*** |
|  | CMP | 2.758*** |
|  | CDP | 6.998*** |
|  | CTP | 6.226*** |
|  | GMP | 3.166*** |
|  | GDP | 4.806*** |
|  | GTP | 4.664*** |
|  | IMP | 10.101*** |
|  | UMP | 2.340*** |
|  | UDP | 5.355*** |
|  | UTP | 4.634*** |
| Deoxy nucleoside phosphates | dADP | 4.969** |
|  | dATP | 7.394*** |
|  | dCTP | 8.728*** |
|  | dGTP | 8.718*** |
|  | dTMP | 9.817*** |
|  | dTDP | 8.185*** |
|  | dTTP | 10.111*** |
| TCA cycle | Cit | 2.351*** |
|  | Fum | 2.713*** |
|  | IA | -1.177*** |
|  | Icit | 0.728** |
|  | aKG | 0.569** |
|  | Mal | 2.555*** |
|  | Suc | -0.380* |
| Amino acids | Ala | 3.852*** |
|  | Arg | 1.295 |
|  | Asn | 2.417* |
|  | Asp | 1.861 |
|  | Cys | 2.333* |
|  | Gln | 4.134** |
|  | Glu | 4.779** |
|  | Gly | 3.507*** |
|  | His | 1.615** |
|  | Ile | 1.487* |
|  | Leu | 1.386* |
|  | Lys | 1.225** |
|  | Met | 1.428* |
|  | Phe | 1.505*** |
|  | Pro | 4.444** |
|  | Ser | 1.600* |
|  | Thr | 2.200** |
|  | Trp | 1.315*** |
|  | Tyr | 1.569*** |
|  | Val | 1.457** |


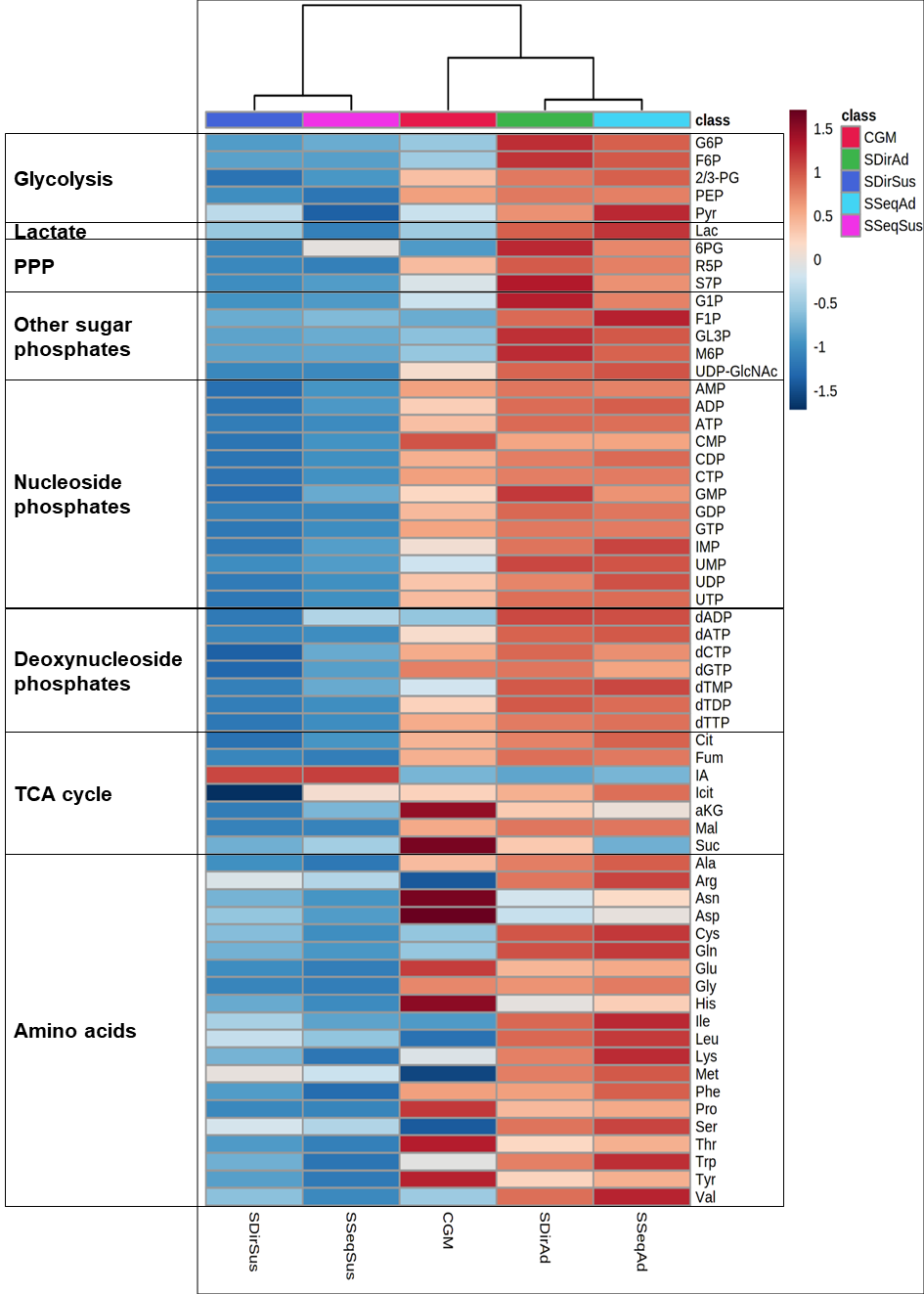


**Supplementary Figure 1.** Heat map of normalized intracellular metabolites pools in 5 different culture systems. The graph was illustrated using Metaboanalyst 5.0. (CGM; Control growth medium culture*,* SSeqAd; Serum-free medium sequential adapted adherent culture, SDirAd: Serum-free medium direct adapted adherent culture, SSeqSus; Serum-free medium sequential adapted suspension culture, SDirSus; Serum-free medium direct adapted suspension culture)


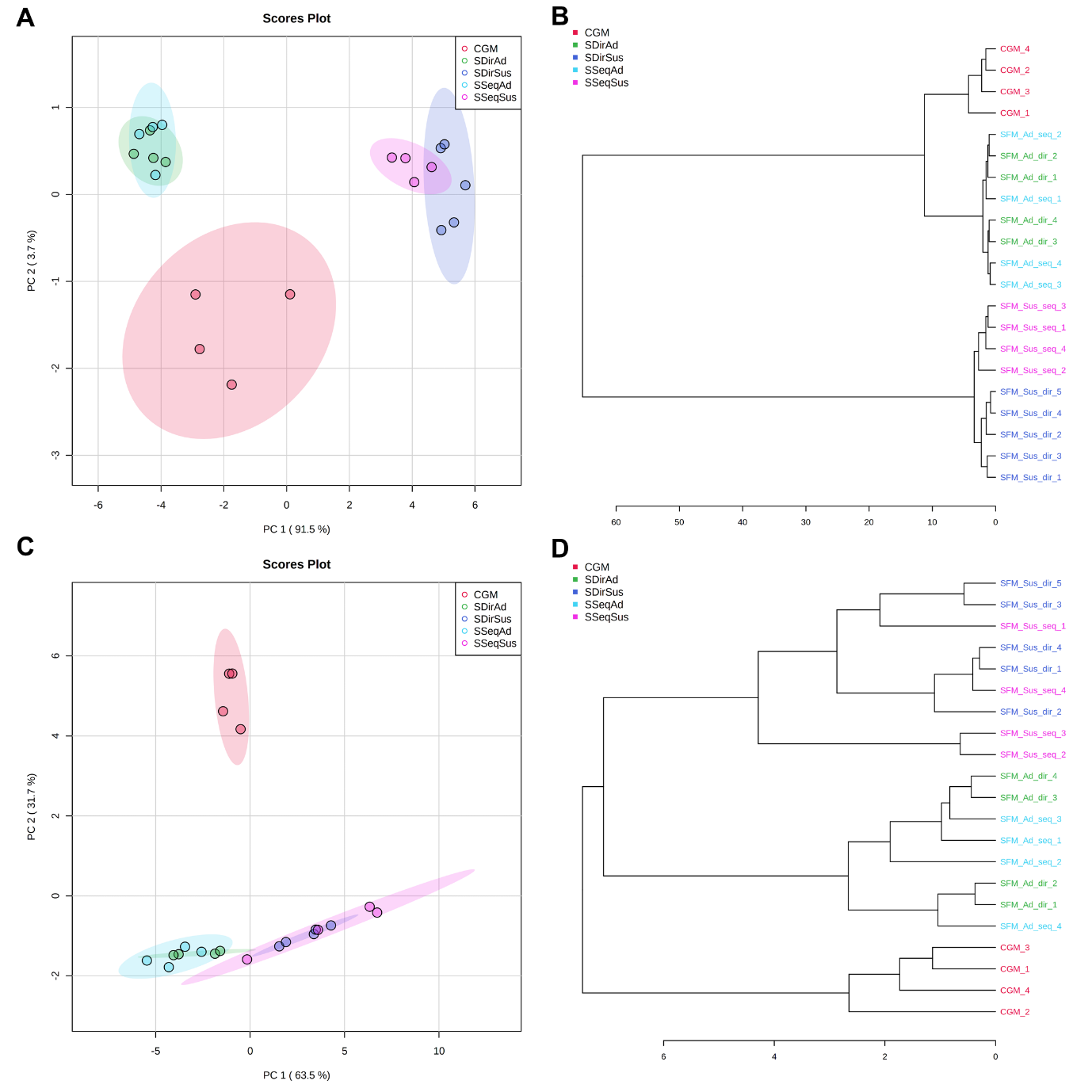


**Supplementary Figure 2.** (A) Principal component analysis (PCA) score plot and (B) Hierarchical cluster analysis (HCA) with nucleotide pools including nucleoside phosphates and deoxynucleoside phosphates. (C) Principal component analysis (PCA) score plot and (D) Hierarchical cluster analysis (HCA) with amino acids pools in 5 different culture groups. (CGM; Control growth medium culture*,* SSeqAd; Serum-free medium sequential adapted adherent culture, SDirAd: Serum-free medium direct adapted adherent culture, SSeqSus; Serum-free medium sequential adapted suspension culture, SDirSus; Serum-free medium direct adapted suspension culture)
